# The natural history of early hepatitis C virus evolution; lessons from a global outbreak in human immunodeficiency virus-1-infected individuals

**DOI:** 10.1099/vir.0.033910-0

**Published:** 2011-10

**Authors:** Emma C. Thomson, Jennifer A. Smith, Paul Klenerman

**Affiliations:** 1 Peter Medawar Building for Pathogen Research, University of Oxford, South Parks Road, Oxford OX1 3SY, UK; 2 Department of Hepatology, Imperial College London, Norfolk Place, London W2 1PG, UK

## Abstract

New insights into the early viral evolution and cellular immune response during acute hepatitis C virus (HCV) infection are being gained following a global outbreak in human immunodeficiency virus-1 (HIV)-positive men who have sex with men. Cross-sectional and longitudinal sequence analysis at both the population and individual level have facilitated tracking of the HCV epidemic across the world and enabled the development of tests of viral diversity in individual patients in order to predict spontaneous clearance of HCV and response to treatment. Immunological studies in HIV-positive cohorts have highlighted the role of the CD4^+^ T-cell response in the control of early HCV infection and will increase the opportunity for the identification of protective epitopes that could be used in future vaccine development.

## Introduction

Studies of the natural history of acute hepatitis C virus (HCV) infection have been restricted in number due to the silent nature of early infection. Insights have been gained from studies in humans and the chimpanzee model, but these have been limited in size due to the lack of availability of subjects and restrictions on the use of primates in laboratory experiments. The global rapid rise of acute HCV in human immunodeficiency virus-1 (HIV-1)-infected men who have sex with men (MSM) is providing the opportunity to study the natural history of acute HCV infection in greater detail and on a larger scale than has been possible previously. Understanding the determinants of spontaneous clearance and response to treatment is important and may provide opportunities for vaccine development as well as new treatment strategies. While the impact of HIV on the evolution of HCV should not be underestimated, new insights from studies in the HIV-positive population are likely to apply also to HIV-negative patients with HCV. We review advances in the understanding of the evolution of HCV in HIV-infected patients at a population and individual level and discuss how further knowledge may be gained from the ongoing study of such patient cohorts.

## Acute HCV in HIV-positive men; a global epidemic

**Fig. 1.**
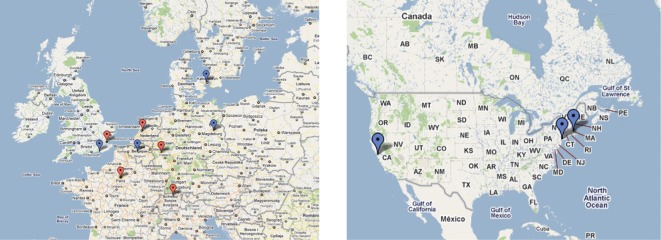
Geographical distribution of acute HCV in HIV-positive MSM in Europe and the USA. A map of Northern Europe and USA showing cities where acute HCV cohorts have been reported. Centres where infection was reported between 2004 and 2005 are shown in red, while those reported in 2006 or later are highlighted in blue.

Reports of sexually transmitted acute HCV infection occurring in HIV-positive MSM in The Netherlands, France, Germany, Switzerland and the UK first appeared in the scientific literature between 2004 and 2005 (Ruys *et al.*, 2004; Ghosn *et al.*, 2004; Vogel *et al.*, 2005; Rauch *et al.*, 2005; Gilleece *et al.*, 2005). Subsequently, outbreaks have been reported in other Northern European countries (Peters *et al.*, 2006; Bottieau *et al.*, 2010) as well as the East and West coasts of the USA (Luetkemeyer *et al.*, 2006; Fierer *et al.*, 2008), and Australia (Nguyen *et al.*, 2007). In total, more than 1000 cases of acute HCV in HIV-positive MSM have been reported worldwide (Vogel *et al.*, 2011). Such patients are commonly asymptomatic and are identified by routine screening at HIV follow-up clinics (Fig. 1).

This remarkable rise in the incidence of acute HCV in HIV-positive MSM is continuing and is likely to herald a serious health burden over the coming years. Already, infection with HCV is emerging as a major cause of morbidity and mortality in individuals infected with HIV and is set to surpass AIDS-defining illnesses as a cause of death in patient populations who have access to highly active antiretroviral therapy (HAART; Bica *et al.*, 2001; Arnold *et al.*, 2006). In Amsterdam, before 2000, the prevalence of HCV in HIV-positive MSM was 1–4 %, while in 2007–2008, the prevalence was 15–21 % (Urbanus *et al.*, 2009). In London, 10 % of MSM HIV seroconverters recruited to an HIV treatment study (SPARTAC) subsequently became infected with acute HCV over a 5 year period (Fox *et al.*, 2008).

Phylogenetic analysis of HCV strains obtained from such patients has shown that a number of monophyletic lineages exist, distinguishing infections in HIV-positive MSM from other risk groups. These lineages cross international boundaries revealing networks of infection occurring in urban centres across Europe and Australia (van de Laar, 2009a). Surprisingly, while intravenous drug use is a risk factor in a minority of cases within each cohort, statistically, the strongest risk factors for transmission of HCV are unprotected anal sex and the use of permucosal (but not intravenous) recreational drugs (Danta *et al.*, 2007; Fierer *et al.*, 2008; Matthews *et al.*, 2009). Preceding medical instrumentation or sexual practices that result in rectal bleeding are particularly strongly associated with transmission (Schmidt *et al.*, 2011).

Molecular clock data points to the origins of the outbreak occurring in the mid-1990s (Danta *et al.*, 2007; de Bruijne *et al.*, 2009). This may reflect a change in sexual risk behaviour and recreational drug use around this time. Most cohorts include a number of individuals who are intravenous drug users (IDUs); although these individuals are in the minority, it is likely that transmission events between IDU and MSM populations have occurred, resulting in a new ecological niche of sexually transmitted HCV in HIV-positive MSM. A feature of the European outbreak has been a high prevalence of genotype 4d HCV (Serpaggi *et al.*, 2006; de Bruijne *et al.*, 2009). Genotype 4 HCV (HCV-4) is the most common circulating strain in North Africa and the Middle East with a decreasing geographical prevalence in countries further north. In Southern European countries, HCV-4 infection is less prevalent but occurs in migrants from North Africa and in IDUs, while in Northern Europe this genotype was previously rare but is increasingly associated with the HIV-positive MSM outbreak. In Amsterdam, two phylogenetically distinct populations of genotype 4d-infected individuals exist; Dutch IDUs and HIV-positive MSM. Molecular clock analysis has shown that the emergence of genotype 4d HCV in IDUs between 1960 and 1990 coincides with increasing recreational drug use in The Netherlands associated with permissive drug legislation (de Bruijne *et al.*, 2009). Later expansion of a subcluster of HCV-4d strains in HIV-positive MSM from 1996 onwards coincides with the availability of HAART and may reflect an increase in unprotected sexual contact due to a reduction in fear of developing AIDS-related illnesses (Stolte *et al.*, 2004). Thus, some of the earliest transmission events from IDUs into MSM may have occurred in Amsterdam. Similar events are likely to have taken place also in other urban centres such as London, Berlin, Paris, San Francisco, New York and Sydney. Strains obtained from patients in Sydney, for example, are largely distinct from those identified in the European outbreak (van de Laar *et al.*, 2009a).

At present, the outbreak of acute HCV appears to be mainly confined to HIV-positive MSM. However, further transmission into other patient groups, for example HIV-negative MSM or heterosexual individuals could provide a potential public health problem in the future. Furthermore, if transmission continues at a high rate in this population, transmission of drug-resistant variants could reduce the future success rate of therapeutic interventions.

## Acute HCV; evolution within the host

### Viral load (VL) dynamics in humans and chimpanzees

**Fig. 2.**
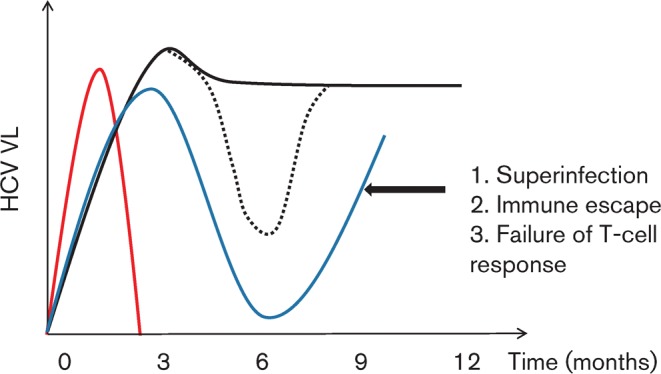
Patterns of viraemia during acute HCV infection. Three patterns of viraemia are seen in acute HCV; spontaneous clearance (red; 15 %) or progression towards chronicity associated with either a plateau viraemia (PV; black; 45 %) or fluctuating viraemia (FV; blue 40 %). Patients with PV may also have FV at an unsampled time point (dashed black line).

A prominent feature of early infection with HCV in both humans and chimpanzees is a peak and then dip in VL (Fig. 2). During spontaneous clearance, this downward descent continues rapidly until viraemia is no longer detectable. In contrast, progression to chronicity is associated with recrudescent viraemia (Dahari *et al.*, 2005; Thomson *et al.*, 2011; Loomba *et al.*, 2011). In human studies, a dip is not always seen. This may not always occur or could reflect the length of sampling windows used to measure sequential VLs in human studies.

VL dynamics can be monitored in order to predict outcome in patients with acute HCV. Chimpanzees experimentally infected with the H77 HCV strain (genotype 1a) had a lower peak VL and a shorter time to peak viraemia in evolving spontaneous clearance than progressing infection. In humans, viral clearance usually occurs within 3 months of diagnosis in both HIV-infected and uninfected cohorts (Santantonio *et al.*, 2006; Page *et al.*, 2009). In a UK-based cohort of 112 HIV-positive patients, the maximum drop in HCV VL within 100 days of the first positive test was strongly predictive of spontaneous clearance [odds ratio (OR) per log_10_ drop = 1.78, *P*<0.0001; Thomson *et al.*, 2011]. Patients who subsequently cleared infection had an earlier and lower peak HCV VL and a steeper gradient of descent than evolving progressor patients, even if the VL became transiently negative. The fluctuating viraemia pattern observed during early HCV infection could represent loss of immune control of circulating virus due to viral escape, superinfection with new strains and/or failure of the T-cell immune response. Studies employing longitudinal viral sequence analysis and T-cell function during early HCV infection in HIV-positive MSM are providing new information about the mechanisms that define continued decrease or increase in VL at early pivotal time points.

### Viral diversification within the host

HCV replicates rapidly (the estimated daily production of virus is 10^12^ HCV viral copies in an infected host) and the viral RNA polymerase does not have proof-reading capacity so the possibility of variation within the HCV genome is extremely high (Neumann *et al.*, 1998). The resulting viral quasispecies consists of a rapidly expanding group of related but genetically distinct viral variants. During early infection, viable viral variants are subject to selection pressures from the innate and acquired immune system as well as other growth restraints such as variation in the concentration of circulating fats (Bader *et al.*, 2008; Felmlee *et al.*, 2010).

Viral genetic sequence analysis can be used to estimate the nature of the prevailing evolutionary force at any point in time. Genetic distance (the percentage difference in nucleotides between quasispecies strains) and measures of diversity such as the Hamming distance (the mean number of amino acid differences between strains) may be used to quantify the composition of the quasispecies population. Selection pressure can be quantified by examining the ratio of non-synonymous substitutions per non-synonymous site (*d*_N_) to synonymous substitutions per synonymous site (*d*_S_); the *d*_N_/*d*_S_ ratio. HCV may be subject to positive (*d*_N_/*d*_S_ >1), purifying (*d*_N_/*d*_S_ <1) or neutral (*d*_N_/*d*_S_ = 1) selection pressures and these may change at different times during infection. The *d*_N_/*d*_S_ ratio model relies on the assumption that silent synonymous mutations are not subject to selection pressure. While these do not result in amino acid changes, regions of the genome that display synonymous variation may have secondary non-coding functions. HCV core and NS5B RNA, for example, contain multiple stem–loops that are found in all HCV genotypes throughout both regions, as well as several strikingly conserved unpaired regions, one of which coincides with a region of the genome to which ribosomal access is required for translation initiation (Tuplin *et al.*, 2004). Variation within the HCV genome is currently considered to be predominantly clonal. Viable recombinant strains has been identified (Kalinina *et al.*, 2002) and the presence of recombinant and parent strains in individual patients have also been reported (Sentandreu *et al.*, 2008). Thus in a minority of cases, recombination events may also contribute to viral diversity.

Genetic variation of HCV is most evident in the hypervariable region 1 (HVR-1), a 28 aa structure within the HCV E2 envelope protein (Weiner *et al.*, 1991) Overall, the rate of nucleotide substitution in HCV is 1×10^−3^ nt per site per year and within the HVR-1 of the envelope gene, this is as high as 2.5–6.9×10^−3^ nt per site per year (Gray *et al.*, 2011) The E2 gene is often targeted for the analysis of viral structure for this reason. Selection within this region is largely antibody-mediated (Farci *et al.*, 2000; Gaud *et al.*, 2003). It also contains CD4^+^ and CD8^+^ T-cell epitopes (Shirai *et al.*, 1999; Lauer *et al.*, 2002; Sarobe *et al.*, 2006). Changes within this region may also reflect CD4^+^ or CD8^+^ cell-mediated variation in regions outwith envelope such as the non-structural genes through a genetic hitchhiking effect or antibody-mediated variation following failure of the cellular immune response (Klenerman *et al.*, 2000).

## The early evolution of HCV

During early infection with HCV, most newly formed viral variants contain potentially detrimental mutations and are subject to strong selection constraints. As a result, purifying selection (*d*_N_/*d*_S_ <1) is the major force moulding viral population structure at this time in HIV-negative (Farci *et al.*, 2000; Kuntzen *et al.*, 2007) and HIV-positive individuals (Thomson *et al.*, 2011). Swinging shifts in viral quasispecies structure are evident as the virus adapts to the environment within a newly infected host (Smith *et al.*, 2010; Thomson *et al.*, 2011). Progression to chronicity is associated with a switch from purifying to positive selection as well-adapted variants outgrow constrained ones. During spontaneous clearance, in contrast, this switch to positive selection does not occur.

The evolution of the viral quasispecies is emerging as key in defining the outcome of infection and response to treatment. Small studies of acute HCV infection have shown that the evolution of HVR-1 is associated with progressive disease, while a narrowed quasispecies repertoire occurs in patients who subsequently spontaneously clear infection due to the gradual elimination of viral variants (Farci *et al.*, 2000; Sheridan *et al.*, 2004; Thomson *et al.*, 2011). In contrast, patients that progress to chronicity exhibit higher viral diversity secondary to viral variation driven by a partially effective immune response. Patients with immunodeficiency; such as a low CD4 count (<200 cells mm^−3^) or agammaglobulinaemia exhibit lower genetic diversity, however, due to a lack of positive immune selection pressure (Gaud *et al.*, 2003; López-Labrador *et al.*, 2007).

## Is spontaneous clearance defined by the transmission bottleneck? Superinfection versus varying dominance during early infection

**Fig. 3.**
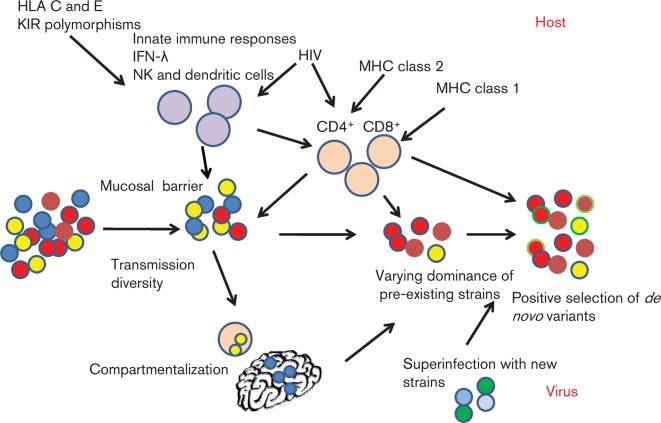
Host and virus factors defining progression to chronicity or spontaneous clearance of acute HCV. The early evolution of HCV is shaped by an arms race between the host and virus. Viral diversification occurs as a result of varying dominance of pre-existing strains and immune selection of *de novo* variants formed during inaccurate viral replication and is aided by replication within compartments such as the brain and PBMCs.

During transmission, infection may be initiated by a single or multiple infectious particles. Measures of viral diversity around this time could potentially be employed to predict the individual risk of progressing to chronicity. However, simple calculations of genetic diversity and positive or negative selection are confounded by the fact that viral diversification during early HCV infection is a non-linear process characterized by superinfection, varying dominance, compartmentalization as well as *de novo* immune escape mutations (Fig. 3).

HIV-infected patients with sexually acquired HCV are more likely to spontaneously clear infection than those infected via intravenous drug use (Shores *et al.*, 2008). In this multicentre study of 769 HIV-infected individuals, patients with a history of sexual transmission spontaneously cleared the HCV infection more often than injection drug users (21.9 versus 11.6 %; *P* = 0.004). This supports the hypothesis that individuals who acquire HCV sexually are subject to a smaller, less diverse inoculum, possibly due to local effects at the mucosal level, although the effect of recreational drugs on the immune system could also partly explain this difference.

Clonal sequence analysis has provided clear evidence for the transmission of multiple HCV strains in both IDUs and sexually infected HIV-positive MSM (Pham *et al.*, 2010; Smith *et al.*, 2010; Thomson *et al.*, 2011). Much of the early variation seen in infected individuals may be explained by varying dominance of strains acquired at the point of transmission. In the chimpanzee model, variation within the HVR-1 region is largely due to the selection of pre-existing variants rather than *de novo* mutations (Fernandez *et al.*, 2004). In humans, this is also the case; in a study of 10 patients predominantly infected by IDU, major shifts in viral diversity occurred with the varying dominant strains (often of different genotypes) switching to and fro over short periods of time (Smith *et al.*, 2010). Both sequential replacement by previously undetected variants and a dominance of variants seen earlier during infection, but not detected in intervening samples, were responsible for this phenomenon. Similar findings were evident in a longitudinal study of 50 HIV-1-infected MSM in which the same primer sets were used (Thomson *et al.*, 2011) and in a cohort of 59 IDU seroconverters (13 of whom were HIV infected) (van de Laar *et al.* 2009b). In the former study, fluctuating changes in viraemia were significantly associated with evidence of superinfection or switching of dominant strains in 63 % of cases. Thus, a diverse infecting population structure is not uncommon and may at least in part define the final outcome of infection.

The narrower quasispecies repertoire evident in those who subsequently spontaneously clear the virus is in keeping with the hypothesis that transmission diversity defines the final outcome. However, using clonal sequence analysis, viral diversity at the earliest sampled time point in a new host does not reliably predict outcome. In a study of 12 HIV-negative individuals with acute HCV, higher diversity was seen only after seroconversion in evolving progressors (Farci *et al.*, 2000) and in a cohort of 50 HIV-positive patients, higher diversity was evident only after two sequential samples taken within 150 days of infection were analysed (Thomson *et al.*, 2011). These results may be biased by the limitations of clonal analysis which may underestimate true viral diversity. Using this technique, variants may be missed that are present in small numbers at transmission (<5 % if 20 clones are sampled at a single time point). Other approaches that are more sensitive than clonal analysis such as pyrosequencing and strain-specific PCR may in the future increase the detection of minority variant strains in baseline samples that later become dominant. In the previously described study of HIV-positive MSM with acute HCV, 36 % of patients who were labelled as superinfected using clonal analysis had a positive PCR result for the apparently superinfecting strain at the onset of infection when tested with strain-specific primers (Thomson *et al.*, 2011). Studies employing pyrosequence analysis may also be useful in the future for distinguishing reinfection with new strains from the emergence of a pre-existing minority variant. However, based on current evidence, it is likely that superinfection and varying dominance of strains both play important roles during early HCV infection.

## Compartmentalization

Detection of minority variants is further compounded by the fact that viral strains may also be compartmentalized and replicate in peripheral blood mononuclear cells (PBMCs; Maggi *et al.*, 1997; Roque Alfonso *et al.*, 1999) and the central nervous system (Forton *et al.*, 2004). Compartmentalization of PBMC-specific strains occurs commonly in HIV-infected individuals with distinct genetic sequences from those detected in plasma (Blackard *et al.*, 2007). Some of the virus detected in such patients may in fact be attached to the surface of NK cells, monocytes and B-cells and be dispersed via this route (Natarajan *et al.*, 2010).

## Predicting clinical outcome using viral diversity measures

Higher viral diversity is likely to increase the risk of progression to chronicity as well as lack of response to treatment due to an increased prevalence of escape mutations and resistant strains.

The point at which such diversity arises after acute infection has not been fully defined but is significantly different in evolving progression and spontaneous clearance by 150 days of infection (Thomson *et al.*, 2011). Those with low Hamming distance and *d*_N_/*d*_S_ ratios are significantly more likely to spontaneously clear the virus than those with high measures of genetic diversity.

Acute HCV is curable in the majority of HIV-negative patients but is less easy to treat in HIV-positive individuals (Jaeckel *et al.*, 2001; Gilleece *et al.*, 2005; Serpaggi *et al.*, 2006; Dominguez *et al.*, 2006). Early diagnosis and treatment of HIV-infected patients with peginterferon (IFN)-α and ribavirin results in improved sustained virological response (SVR) rates [59 % in acutely versus 40 % in chronically infected patients (Gilleece *et al.*, 2005; Torriani *et al.*, 2004)], but does not reach the 98 % treatment success rate reported in HIV-negative individuals (Jaeckel *et al.*, 2001). The reason for this difference is not fully understood but could be partially explained by a higher rate of viral evolution and the formation of multiple variants due to HIV-related impaired immune control.

In keeping with the viral diversity theory, studies in HCV monoinfection have shown that two regions of the genome correlate with the likelihood of SVR in patients with chronic HCV (genotypes 1 and 3); the E2 HVR-1 and the IFN sensitivity determining region (ISDR) within NS5A (Enomoto *et al.*, 1995; Enomoto *et al.*, 1996; Ueda *et al.*, 2004; Shire *et al.*, 2006; Moreau *et al.*, 2008). Increased diversity within E2 is associated with lower SVR. The mean number of mutations within the ISDR that differ from resistant prototype genotype 1a, 1b and 3a strains increases the likelihood of SVR (a mean two to four mutations difference confers protection; Pascu *et al.*, 2004; MacQuillan *et al.*, 2004; Nakagawa *et al.*, 2010). A mutation within HCV core – the R70Q substitution is also strongly associated with resistance and lack of early virological response (EVR; Enomoto & Maekawa, 2010). The role of these resistance markers in acute infection and in HIV-infected individuals is as yet unknown.

## Viral diversity is governed by the immune response

The key role of both innate and acquired immune responses in the control of early HCV infection is supported by a large body of evidence (recently reviewed by this journal; Jo *et al.*, 2011). Different areas of the viral genome are subject to different selection pressures; the E2 HVR-1 is largely subject to antibody responses, while core and the non-structural proteins are moulded by the CD4^+^ and CD8^+^ acquired immune response.

## Innate immune responses

While the key role of innate immunity in controlling HCV infection is well established, the impact of innate responses on viral diversity remains relatively unexplored. Single nucleotide polymorphisms near or within the IL-28B locus (the gene that encodes IFN-λ3) are associated with spontaneous clearance and response to treatment in HIV-positive and -negative individuals (Thomas *et al.*, 2009; Tanaka *et al.*, 2009). It is not clear yet whether IFN-λ encoded by this region is responsible for such effects. Among HIV-infected patients with chronic HCV, those bearing the IL-28B genotype CC were more commonly infected with genotype 3 than subjects with non-CC genotypes; thus, the protective effect of the CC genotype is mainly exerted in patients infected with HCV genotypes 1 or 4 (Neukam *et al.*, 2011). While favourable IL-28B polymorphisms are associated with SVR in chronically infected HIV-positive patients, they are not associated with SVR in acute HCV (Nattermann *et al.*, 2011).

Natural killer (NK) cell responses are also strongly associated with outcome during acute HCV (recently reviewed by Cheent & Khakoo, 2011); for example, spontaneous clearance is associated with the presence of the inhibitory receptor KIR2DL3 and the HLA-C1 ligand. This gene combination may be protective because the KIR2DL3 binds HLA-C with a lower avidity than other inhibitory KIRs, and thus NK cells expressing this specific inhibitory receptor have a lower threshold for activation.

Studies investigating the impact of such host genetic polymorphisms on viral diversification are awaited.

## Acquired immune responses

The impact of both B- and T-cell responses on viral diversity is well-established. Immune pressure may result in swings in dominant quasispecies and the selection of HLA-defined epitopes. The role of individual epitopes in defining the final outcome is less clear.

## B-cell responses

As discussed above, a gradual increase in genetic diversity and *d*_N_/*d*_S_ ratio in the E2 HVR-1 gene is likely to be mediated predominantly by antibody responses (Farci *et al.*, 2000; Gaud *et al.*, 2003). High-resolution analysis of positively selected codons during early HCV infection show that these gradually accumulate in progressors (Sheridan *et al.*, 2004) but not in clearers. Low CD4 count in HIV-positive patients is also associated with lower HVR-1 diversity, while higher CD4 counts following treatment of HIV with HAART is associated with an increase in HVR-1 diversity (Bernini *et al.*, 2011). This may not be protective; the HVR-1 could be a decoy site; increasing diversity within the HVR-1 in this study was also significantly associated with an increase in HCV VL.

## T-cell responses during co-infection with HCV and HIV

Both CD4^+^ and CD8^+^ responses are critical in defining the final outcome of HCV infection. Evidence for this comes from the chimpanzee model, epidemiological HLA-association studies and functional and phenotypic studies of T-cells in acutely infected individuals. Protective immunity in HIV-negative individuals is known to be associated with multi-specific CD4^+^ and CD8^+^ T-cell responses (Cooper *et al.*, 1999; Lechner *et al.*, 2000; Harcourt *et al.*, 2001; Cox *et al.*, 2005). In patients with chronic HCV, both the magnitude and breadth of CD4^+^ and CD8^+^ responses are lower in HIV-infected patients (Capa *et al.*, 2007). The role of CD4^+^ T-cells is likely to be of particular relevance in patients who are HIV infected.

In chimpanzees, depletion of CD4^+^ cells results in immune escape and persistent infection (Grakoui *et al.*, 2003). In humans, MHC class 2 associations also provide evidence for the role of CD4^+^ cells in spontaneous clearance; HLA DRB1*0101, DR1B1*1101 (OR = 2.14) and DQB1*0301 (OR = 2.22) are associated with spontaneous resolution of acute HCV (Thursz *et al.*, 1999) and an improved immune response (Harcourt *et al.*, 2001), while HLA-DRB1*0701 is associated with viral persistence (Fanning *et al.*, 2001).

As might be expected, CD4^+^ responses to acute HCV are weaker in HIV-positive individuals. In particular, significantly reduced CD4^+^ IFN-γ responses occur in HIV-positive patients, especially those directed against NS3-5 (Danta *et al.*, 2008). Targeting of NS proteins is associated with spontaneous clearance in both HIV-negative and HIV-positive individuals, while responses to core are more common in chronically evolving infection (Gerlach *et al.*, 2005; Aberle *et al.*, 2006; Ruys *et al.*, 2008; van den Berg *et al.*, 2009; Schnuriger *et al.*, 2009).

A key role of CD4^+^ cells is to provide support for CD8^+^ T-cells and CD4^+^ cell dysfunction impacts on the CD8^+^ response in both chimpanzees and humans. CD4^+^ T-cell help is critical in maintaining long-term protective CD8^+^ T-cell immunity in chimpanzees (Zajac *et al.*, 1998; Grakoui *et al.*, 2003) and in humans (Urbani *et al.*, 2006). In humans, a cross-sectional analysis of HCV-specific CD8^+^ cell responses during chronic HCV and HIV infection revealed that HIV-related CD4 cell depletion was associated with significantly lower HCV-specific CD8^+^ responses (*P*<0.0001; Kim *et al.*, 2005).

This is important, as CD8^+^ T-cells also play a key role in HCV outcome. Depletion of CD8^+^ cells in chimpanzees is associated with viral persistence (Shoukry *et al.*, 2004). In humans, MHC class 1 associations (particularly HLA B27) with spontaneous clearance provide evidence of the role of the CD8^+^ response in defining outcome (McKiernan *et al.*, 2004; Neumann-Haefelin *et al.*, 2007). Footprints of the HLA-defined T-cell response (HLA-defined viral escape mutations from CD8^+^ T-cell responses) may be found within the viral quasispecies in the HCV structural and NS proteins (Cox *et al.*, 2005). The number of mutations occurring in the viral population at any time due to CD8^+^ selection pressure is as much as 32 %; this is significant, but lower than that observed in early HIV infection (30–60 %; Pfafferott *et al.*, 2011). This figure is likely to be lower in HIV-infected individuals as HCV-specific CD8^+^ responses are weaker (Capa *et al.*, 2007). Interestingly, reversion from HLA-restricted epitopes does not appear to be common in HCV following transmission. This may be due to the co-existence of deleterious and compensatory mutations in differing parts of the viral genome. Such epistatic mutations are evident in HCV cell culture and in natural infection; thus evolution of HCV is constrained not just by the immune response but also by other fitness considerations and evolution of the genome occurs in a co-ordinated fashion (Campo *et al.*, 2008).

## Summary and future perspectives

The emerging epidemic of acute HCV in HIV-positive MSM has allowed the study of acute HCV infection on a large scale. Studies of viral evolution have shown that immune control of infection is hampered by rapid diversification due to switching dominance of viral quasispecies, superinfection, generation of *de novo* escape variants and compartmentalization of the virus. The key role of the CD4^+^ response in controlling infection is highlighted in HIV-positive cohorts. Many questions remain unanswered however. Firstly, the predictive role of baseline viral diversity in defining spontaneous clearance may be increased using techniques in which viral genetic diversity may be assessed in greater depth. Such analyses may also assist in predicting the response to treatment. The time at which the quasispecies diversity reaches a threshold level may define the suitability and timing of treatment and with the advent of newer treatments for HCV such as the protease inhibitors bocepravir and telapravir could potentially be used to target treatment for appropriate individual patients. Finally, the availability of larger cohorts of patients may facilitate the definition of particular protective epitopes which may be used in future vaccine development.
